# Key factors influencing undergraduate nursing students’ perceptions of the use of learning management systems: a systematic literature review

**DOI:** 10.1186/s12912-025-02962-9

**Published:** 2025-03-26

**Authors:** Fathiya Alkhuzaimi, Christine Brown Wilson, Wai Yee Amy Wong

**Affiliations:** 1 School of Nursing and Midwifery, Queen’s University Belfast, University Road, Belfast, BT7 1NN Northern Ireland; 2 Present Address: Norwich Medical School, University of East Anglia, Norwich, UK

**Keywords:** Learning management system, Nursing students, Perception, Experience, Blended learning

## Abstract

**Background:**

The technological revolution has significantly transformed educational practices, particularly through the implementation of learning management systems (LMS). Understanding the perspectives of undergraduate nursing students regarding the use of LMS is essential, as these perceptions can significantly influence their learning experiences and outcomes. This systematic review aims to identify and explore the factors influencing these students’ perceptions of LMS.

**Methods:**

A systematic review was conducted by searching five electronic databases—CINAHL Plus, Medline, Embase, Web of Science, and the Cochrane Library—for studies published between 2010 and 2020. An updated search was performed in July 2024 to ensure the inclusion of recent evidence. Studies were screened against predefined inclusion and exclusion criteria, focusing on undergraduate nursing students and their experiences with LMS. The included studies utilised a range of designs: mixed-methods (4), cross-sectional (3), quantitative descriptive surveys (5), randomised controlled trials (1), qualitative (2), case studies (1), quasi-experimental (2), and observational (1). The quality of the studies was rigorously assessed using the Critical Appraisal Skills Programme (CASP), Milton Keynes Primary Care Trust (MKPCT) tools, the Mixed Methods Appraisal Tool (MMAT), and Joanna Briggs Institute (JBI) tools. Data were synthesised through thematic analysis, following Braun and Clarke’s framework.

**Results:**

In total, 19 studies were included in the review, encompassing a diverse range of research designs. The review identified several factors that significantly influenced students’ acceptance and perception of LMS. These factors included students’ digital literacy, prior experience with technology, motivation, and self-efficacy. Additionally, key organisational factors, such as instructor support and the availability of training, were associated with positive perceptions of the LMS. Specific features of the LMS, including ease of use, interactive elements, and accessibility, also contributed to enhancing students’ perceived ease of use (PEOU) and perceived usefulness (PU).

**Conclusion:**

Students’ perceptions of LMS are closely linked to their acceptance of these platforms, as guided by the Technology Acceptance Model (TAM). Digital literacy, prior technology experience, and self-efficacy emerged as critical factors positively influencing perceived usefulness and ease of use, leading to greater acceptance and satisfaction. Instructor support and interactive LMS features were also vital for enhancing engagement and learning outcomes. These findings underscore the importance of considering these factors in the design of LMS modules for undergraduate nursing students. Future research should investigate the long-term effects of LMS use on learning outcomes to inform best practices.

**Supplementary Information:**

The online version contains supplementary material available at 10.1186/s12912-025-02962-9.

## Background

The recent technological revolution has substantially reshaped the education sector, driving advancements in teaching and learning methods in higher education institutions worldwide [1]. Innovations in information and communication technology (ICT) have enabled higher education institutions to adopt flexible teaching and learning approaches, including e-learning, which leverages digital technologies to support learning beyond traditional classroom settings. E-learning modalities, such as blended learning (BL) and flipped learning, have been shown to enhance learner engagement, flexibility, and knowledge retention, improving the student experience overall [2].

Driven by digital advancements, e-learning strategies such as BL and flipped learning are now integral to modern curricula [3]. The Joint Information Systems Committee (JISC) has documented rapid growth in BL and online learning adoption within higher education institutions in the United Kingdom (UK) [4]. This trend accelerated considerably during the COVID-19 pandemic, as higher education institutions were pressed to rapidly transition from face-to-face (F2F) to online instruction. Institutions with preexisting learning management systems (LMS) were better equipped to manage this shift, leveraging established digital infrastructures to ensure instructional continuity [5]. LMS, therefore, play a crucial role in transforming instructional practices. However, disciplines such as nursing face unique challenges in adapting to online formats because of the essential requirements for clinical placements and hands-on skill acquisition [6]. Accordingly, it is timely to explore how e-learning, especially through LMS, shapes the learning experiences of undergraduate nursing students.

In health profession education, the adoption of BL and e-learning models has gained global traction, particularly in nursing programs following the onset of the COVID-19 pandemic. Many Western countries, including the UK, New Zealand, Greece, Ireland, Canada, the United States of America (USA), and Australia, have incorporated e-learning strategies into their nursing curricula [7]. While e-learning has gained popularity and has shown positive learning outcomes in higher education institutions [8], a notable gap remains in understanding how LMS, specifically, influence nursing students’ academic experiences. Recognising this gap is essential, as LMS are increasingly central to modern nursing programs, where theoretical knowledge and practical skills must align effectively for successful learning outcomes [8]. Therefore, this review seeks to address this gap by examining the key factors that influence undergraduate nursing students’ perceptions of using LMS in their studies.

To systematically explore students’ perspectives on LMS usage, this review applies the technology adoption model (TAM) as a guiding framework. The TAM offers a structured approach for investigating technology adoption within educational settings, rooted in the unified theory of technology adoption and the theory of reasoned action [9]. According to the TAM, two critical factors—perceived usefulness (PU) and perceived ease of use (PEOU)—shape users’ attitudes toward LMS and predict their acceptance of these systems for educational purposes [10]. A meta-analysis of 88 studies confirmed the reliability of the TAM in establishing causal relationships, with perceived usefulness emerging as a strong predictor of technology acceptance [11]. The systematic literature review indicates that the TAM is significant in understanding factors influencing the adoption of LMS, highlighting its utility in exploring users’ intentions, attitudes, and contextual influences [12].

Using the TAM framework, this review aims to provide a comprehensive understanding of the factors shaping undergraduate nursing students’ perceptions of e-learning through LMS. The term “undergraduate nursing students” is used as it is globally recognised, ensuring clarity and consistency across different contexts. While “pre-registration” is commonly used in the UK, this review focuses exclusively on undergraduate nursing students to align with the PICO framework and target the largest segment of pre-registration nursing students worldwide. By doing so, it seeks to contribute valuable insights into the broader discourse on digital education in healthcare, thereby informing the integration of LMS in nursing education to better support student learning and engagement.

### Review question

What are the key factors influencing undergraduate nursing students’ perceptions of using LMS in their studies?

## Method

This systematic literature review followed the PRISMA guidelines for reporting systematic reviews [13]. A systematic review was chosen to rigorously identify, evaluate, and synthesise high-quality evidence on factors influencing undergraduate nursing students’ perceptions of LMS. This approach enabled a comprehensive and structured investigation, ensuring the inclusion of only the most relevant and reliable studies. The method is particularly suitable for generating detailed, evidence-based findings that may contribute to improving educational practices in nursing programs.

### Search strategy

A subject-specific librarian assisted in developing the search strategy, which was informed by the population (P), phenomenon of interest (I), and context (Co) (PICo) framework [14] (Table 1). Although the PICo framework is traditionally associated with qualitative research, it was selected for this review due to its suitability for capturing perceptions and experiences, which align with the research question. This approach allowed for the inclusion of diverse study designs while ensuring consistency in eligibility criteria. Grey literature, including unpublished research, was excluded to focus on peer-reviewed studies with established methodological rigor. This decision aligns with the aim of synthesising high-quality evidence relevant to the research question.

**Table 1:** PICo framework of the current study

P (Population)	Undergraduate nursing students
I (Phenomenon of interest)	Students’ perception of using LMS.
Co (Context)	Universities or nursing colleges where a LMS is incorporated as part of the teaching strategy

A comprehensive search was conducted via a combination of search terms across five databases: EMBASE, CINAHL Plus, Medline, Web of Science, and the Cochrane Library. The search covered studies published from 2010 to 2020, with an updated search performed in July 2024 to capture the latest evidence. This period reflects the timeframe during which LMS became widely adopted in education [4]. Additionally, the reference lists of relevant studies and systematic reviews were screened to identify additional papers. The complete search strategy and results are presented in Supplementary File 1: Tables S1, S2, and S3.

### Eligibility criteria

#### The inclusion criteria were as follows

- The study population was undergraduate nursing students, as this review aimed to explore the perceptions of this specific group in relation to LMS.
- Blended learning (BL) is delivered via the LMS, as the review focused on how LMS-based BL approaches influence undergraduate nursing education.
- Primary studies were published in peer-reviewed journals to ensure the inclusion of high-quality, empirical evidence.
- The publication year ranged from 2010 onwards, reflecting the period during which LMS adoption became widespread, as noted in the search strategy.
- The publication language used was English, with only English-language studies included.

#### The exclusion criteria were as follows

- The study population was not undergraduate nursing students (e.g., postgraduate and doctoral students), as they were not the focus of this review.
- BL not delivered via LMS, as the review focused specifically on LMS-based learning approaches.
- Editorials, commentaries, or discussion papers do not provide empirical evidence.
- Unpublished research and grey literature, as the review focused exclusively on peer-reviewed studies to ensure methodological rigour.

### Data management and selection process

**Fig. 1:**
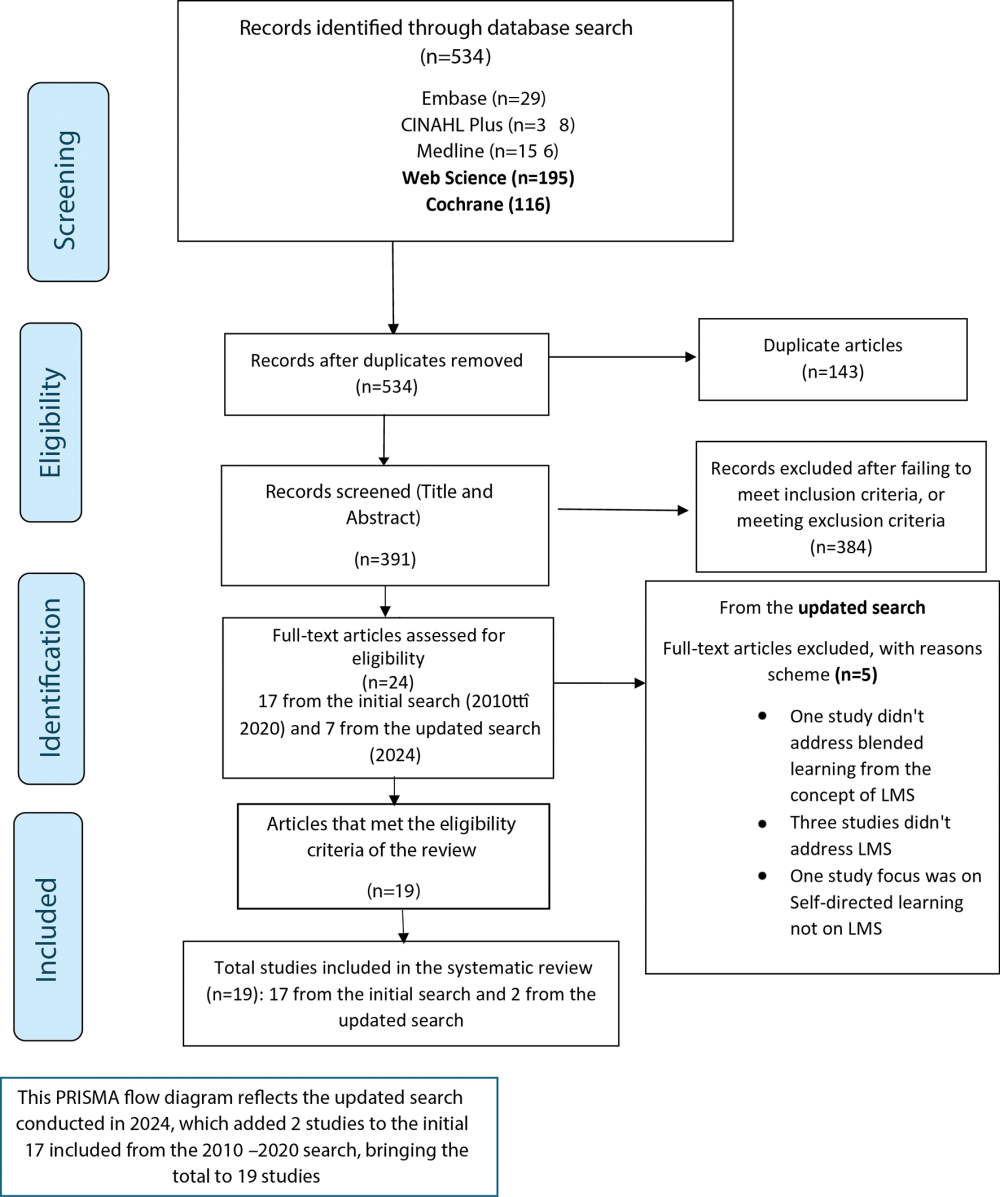
Presents the study’s Updated PRISMA flow diagram for search results (2024). PRISMA flow diagram. Page M, McKenzie J, Bossuyt P, Boutron I, Hoffmann T, Mulrow C, and Moher D. The PRISMA 2020 statement: an updated guideline for reporting systematic reviews. BMJ.2021;372 (71). http://www.prisma-statement.org/. Accessed 15 August 2024

Titles and abstracts retrieved from the database searches were independently screened by three researchers (FA, CBW, and WYAW). In cases of disagreement, the team discussed the studies to reach a consensus. After the initial screening, full-text papers were reviewed against the inclusion and exclusion criteria by the same three researchers to ensure that all included studies addressed the research question. Any disagreements regarding full-text inclusion were resolved through team consensus. The detailed reasons for the exclusion of full-text papers are provided in Supplementary File 1, Table S4. The study selection process is visually summarised via the PRISMA 2020 flow diagram, presented in Fig. 1 of the results section.

### Data extraction

Three reviewers participated in the data extraction process. Data from included studies were first extracted into a table of study characteristics to improve clarity and facilitate analysis. The extracted details included author names, publication year, journal name, study aims, design, outcome measures and instruments, sample size, LMS used, study quality, main findings, conclusions, and limitations. For further details, please see Supplementary File 2, Table S1.

### Quality assessment of the studies

The methodological quality of the included studies was independently assessed by three reviewers (FA, CBW, and WYAW) using four critical appraisal tools tailored to the study designs: the Critical Appraisal Skills Programme (CASP) [15], the Milton Keynes Primary Care Trust (MKPCT) tool [16], the Joanna Briggs Institute (JBI) Critical Appraisal Checklist for Quasi-Experimental Studies [17], and the Mixed Methods Appraisal Tool (MMAT), version 2018 [18]. Each tool was selected to ensure an appropriate and rigorous evaluation of methodological quality. The CASP tool was applied to randomised controlled trials (RCT), qualitative studies, and case studies. This tool assessed aspects such as study design, participant selection, and risk of bias, with studies rated as high, moderate, or low quality based on adherence to these criteria. The MKPCT tool, which is specifically designed for cross-sectional and survey studies, evaluated the reliability and validity of study designs and data collection methods. Studies were categorised as high, moderate, or low quality according to their methodological rigour.

The JBI Critical Appraisal Checklist for Quasi-Experimental Studies was used to assess quasi-experimental studies. This tool evaluated elements such as participant selection, clarity in cause-and-effect relationships, use of control groups, and the reliability of outcome measurements. Each study was classified as high, moderate, or low quality based on its methodological robustness. For mixed-methods studies, the MMAT (version 2018) was used to evaluate both qualitative and quantitative components. The tool examined aspects such as coherence between research questions and data sources, appropriateness of data analysis, and integration of qualitative and quantitative findings. Studies were rated as high, moderate, or low quality based on their overall methodological soundness. Any disagreements between reviewers were resolved through discussion until consensus was achieved. The final quality rating of each study was determined using the criteria set by each respective appraisal tool. Full details of the quality assessments for all included studies are provided in Supplementary File 3, Table S1.

### Data analysis and synthesis

Thematic analysis (TA) was applied in this review to identify key themes and subthemes emerging from the selected studies, following Braun and Clarke’s six-step framework [19]. Reflexive thematic analysis was chosen for its flexibility in synthesising findings across diverse study designs and its ability to provide a nuanced understanding of patterns in the data, aligning with the review’s aim to explore factors influencing students’ adoption of LMS. The synthesis focused on factors influencing the adoption and use of LMS by undergraduate nursing students, guided by the TAM, which provided a structured framework for examining constructs such as PEOU and PU [20]. While the term ‘factors’ was used to describe influencing variables in the context of TAM, the findings are presented as ‘themes’ to reflect the interpretative nature of the data analysis process inherent in TA. The data analysis process began with familiarisation, where all reviewers (FA, CBW, and WYAW) independently reviewed the extracted data to develop an in-depth understanding. Initial codes were then independently assigned by each reviewer, identifying key concepts related to students’ perceptions of LMS, particularly usability and effectiveness. This systematic, initial coding framework was then applied across studies, grouping related codes into broader categories that formed the basis of the subthemes. The coding process was iterative, with regular team discussions to refine the codes and ensure consistency. Codes were further analysed to search for patterns and relationships, leading to the development of key themes that captured common and contrasting findings across the studies. These themes were reviewed and refined collaboratively to ensure alignment with the research questions and the TAM framework. The constructs of PEOU and PU were central to the analysis, helping to contextualise findings within the broader LMS adoption landscape.

Once themes were established, a narrative synthesis was conducted to integrate the findings, involving comparisons and contrasts across studies to highlight similarities and differences. Given the heterogeneity of the studies—such as variations in sample size, study design, and LMS platforms—a narrative synthesis was chosen, as a meta-analysis was not feasible. The data analysis process included a critical examination of the strengths and limitations of the included studies, with a focus on the methodological quality and relevance of the findings. This comprehensive approach offered insights into key factors affecting LMS adoption in undergraduate nursing education and provided recommendations for future research and educational strategies.

## Results

The initial search (2010–2020) identified 528 studies, which were imported into EndNote Software Version X9. After removing 143 duplicates, 385 unique titles and abstracts were screened, yielding 21 full-text studies for further review. Six studies were excluded for not focusing on LMS, leaving 15 studies meeting the inclusion criteria. An additional two studies were identified through reference list screening, resulting in a total of 17 studies.

An updated search conducted in 2024 identified 534 records. After screening, seven studies underwent full-text review, with five excluded for not meeting eligibility criteria. Two additional studies were included, bringing the total number of studies in this systematic review to 19. The updated PRISMA flowchart is presented in Fig. 1.

### Study characteristics

The included studies were conducted across 16 countries, including Australia [29–31], Canada [21], China [33], Egypt [36], Finland [38], France [28], Iran [24, 25], Norway [27], Oman [34], Saudi Arabia [26], Singapore [37], South Africa [23], Spain [32], Sweden [35], Taiwan [39], and the United Kingdom [22]. Various LMS platforms were utilised, including Moodle [23, 32, 34, 39], Blackboard [26, 36], Adobe Connect [31], and the Tsinghua Education Online (THEOL) platform [33]. Ten studies did not specify the LMS used.

Sample sizes ranged from 12 participants in a qualitative study [38] to 1000 participants in an online survey [36]. Twelve studies provided demographic details, reporting female participants accounted for 60–100% of the sample. Four studies did not report participant ages [27, 30, 33, 39], while the remaining studies included participants aged 18–48 years.

### Quality of included studies

The methodological quality of the 19 studies varied and was assessed using appropriate critical appraisal tools. Mixed-method studies [22, 27, 30, 31] and cross-sectional studies [24, 35, 36] were rated as high quality, demonstrating robust designs and rigorous methodologies. Quantitative descriptive surveys [23, 26, 28, 29, 33] displayed varied quality, with four rated as high quality [23, 26, 29, 33], and one [28] rated as moderate to high quality due to recruitment and sample size limitations.

The single RCT [21] was rated as low to moderate quality due to methodological limitations but was deemed locally applicable. The qualitative studies [37, 38] were rated as good quality, reflecting robust data analysis. The case study [39] and quasi-experimental studies [25, 32] exhibited strong methodological adherence and were rated as high quality, as was the observational study [34]. Limitations included small sample sizes and potential biases in some studies. Detailed quality appraisals are available in Supplementary File 3, Table S1.

### Key factors influencing undergraduate nursing students’ perceptions of LMS use

Understanding how undergraduate nursing students perceive LMS is crucial for enhancing their educational experience. Therefore, this analysis sought to answer the following question: What are the key factors influencing these perceptions? The findings reveal three overarching factors that significantly influence undergraduate nursing students’ perceptions of LMS: individual, organisational, and technological factors. A list of the associated factors is identified under each overarching factor, as shown in Table 2.

**Table 2:** Overarching factors and associated factors

Overarching factor	Associated factors
Individual	Demographics, digital literacy, prior technology experience, motivation, self-efficacy, technology acceptance
Organisational	Instructors’ Role, Students’ Training and Support, Technology Access
Technological	Features of LMS

## Overarching factor

### Theme 1: individuals

The studies identified a significant relationship between individual factors and the perceptions of undergraduate nursing students regarding LMS [22–24, 26, 30, 31, 36]. These individual factors encompass students’ demographics, digital literacy, prior experience with LMS, motivation, self-efficacy, and technology acceptance. Collectively, these elements shape students’ engagement with LMS, influencing their perceptions, academic performance, and overall satisfaction with online learning. The following sections explore how each of these individual factors affects students’ interactions with LMS.

#### Students’ demographics (age and gender)

The findings of four studies [23, 24, 26, 36] reported associations between students’ age and their attitudes towards using LMS; however, these associations varied across the studies. Roudsar et al. [24] suggested that students aged 20–21 years presented higher participation levels in LMS than did those aged 24 years and above. Similarly, Chipps et al. [23] reported that students with a mean age of 21.3 years engaged more with LMS than those with a mean age of 40.4 years. In contrast, Elbasuony et al. [26] reported that students aged 22 years and older participated more frequently and accessed LMS more than younger students aged 21 years did. Despite these variations, no consistent significant impact of age on students’ usage of and attitudes towards LMS was found across the studies. Therefore, it appears that students’ age does not exert a direct influence on their perceptions of LMS.

Gender differences were also minimal. Two studies [24, 27] explicitly explored this factor and reported no significant differences in LMS perceptions between male and female students, despite the majority of participants being female. Thus, demographic factors, including age and gender, appear to have a limited influence on students’ perceptions and usage of LMS.

#### Students’ digital literacy

Seven studies [22, 23, 26, 28, 30, 31, 36] identified digital literacy as a key factor influencing undergraduate nursing students’ use and perceptions of LMS. Higher levels of digital literacy were consistently associated with more frequent LMS use and more positive perceptions. Meedya et al. [30], O’Flaherty and Laws [31], and Mousa et al. [36] reported that students with advanced digital literacy not only engaged more with LMS but also expressed greater satisfaction with these systems.

Similarly, Chipps et al. [23] demonstrated a significant relationship between digital skills and ease of LMS use, with students possessing higher literacy levels reporting significantly greater ease of use (*p* = 0.001). Bloomfield and Jones [22] supported these findings, noting that students with strong digital literacy were better equipped to navigate LMS, while those with lower digital literacy encountered technical challenges such as password issues and inconsistent internet access. These barriers were particularly evident as students adjusted to university life.Conversely, students with lower digital literacy expressed dissatisfaction with LMS use, as highlighted by Meedya et al. [30], O’Flaherty and Laws [31], and Marco et al. [28]. Overall, these findings underscore the importance of digital literacy in shaping students’ PEOU and PU of LMS, ultimately influencing their engagement and perceptions of these tools.

#### Students’ prior experience with LMS

Seven studies [22–24, 26, 33, 35, 36] highlighted the significant influence of prior LMS experience on students’ perceptions and academic performance. Renmarker and Carlson [35], through questionnaires administered in semesters one and six, assessed nursing students’ experiences with a web-based drug calculation platform. Their findings revealed a positive association between prior e-learning exposure and favourable perceptions of the LMS, with students evaluating the platform as useful and supportive for learning.

Similarly, Elbasuony et al. [26] reported that 74% of participants (*N* = 80) with prior Blackboard experience expressed significantly higher technology acceptance and positive LMS perceptions, aiding them in meeting module requirements. Supporting these findings, Shang and Liu [33] conducted two surveys at different program stages: an initial survey where 69% of students preferred traditional teaching, and a later survey where 67% preferred e-learning. This shift suggested that experience gained during the semester directly influenced their perceptions of LMS.

Mousa et al. [36] demonstrated a strong positive correlation between Blackboard experience and overall attitude scores, indicating that favourable LMS experiences increased usage frequency and enhanced perceptions. Chipps et al. [23] further reported that 72.1% of participants (*N* = 213) perceived LMS as useful and easy to use due to prior technological experience, which positively impacted their cognitive ability, academic participation, and achievement. Overall, these findings underscore the critical role of prior technological and LMS experience in shaping students’ attitudes, usage, and perceptions of LMS, ultimately enhancing their academic engagement and outcomes.

#### Students’ motivation

Six studies [21, 22, 28, 31, 34, 38] highlighted the significant role of motivation in shaping students’ engagement with LMS, influencing their interaction, participation, and academic outcomes. Motivation was found to enhance self-directed learning, knowledge acquisition, and satisfaction with LMS.

Mäenpää et al. [38] emphasised that active participation and interaction with instructors and peers through LMS were critical for sustaining motivation, fostering positive perceptions of blended learning, and enhancing self-directed learning skills. Similarly, O’Flaherty and Laws [31] reported that 89% of survey respondents (*N* = 101) indicated that engagement with instructors and peers on LMS motivated them to acquire knowledge. Marco et al. [28] found a statistically significant relationship (*p* < 0.001) between motivation and knowledge acquisition through e-learning systems.

Motivation also directly impacted students’ perceptions, engagement, and module achievement. Bloomfield and Jones [22] reported that motivated students achieved outcomes 25% better than their less motivated peers, while Amandu et al. [34] observed a similar trend, with motivated students achieving outcomes 30% better. In a randomised controlled trial, Gagnon et al. [21] noted that motivation positively influenced satisfaction with LMS, with motivated students significantly outperforming their peers in learning outcomes (*p* = 0.0005). Conversely, Meedya et al. [30] highlighted challenges faced by students lacking motivation, including reduced satisfaction and involvement due to the absence of nonverbal communication in LMS. These findings collectively underscore the pivotal role of motivation in enhancing students’ perceptions, engagement, and academic performance when LMS are utilised.

#### Students’ self-efficacy

Four studies [25, 37–39] underscored the importance of self-efficacy in enhancing students’ engagement with LMS. Self-efficacy was consistently linked to greater confidence, active participation, and improved academic outcomes.

Shorey et al. [37] found that active participation in e-learning activities and constructive feedback from peers and instructors strengthened students’ belief in their ability to succeed, fostering ongoing engagement with LMS. Similarly, Mäenpää et al. [38], through interviews with 12 third-year nursing students, reported that those with higher self-efficacy were more likely to set and achieve academic goals, resulting in improved performance and deeper LMS engagement.

Yang and Lin [39], in a case study, demonstrated a significant association (*p* < 0.05) between self-efficacy, attitudes towards Moodle, and LMS engagement. Students with higher self-efficacy actively participated in LMS activities and used the system more effectively.

Malsakpak and Pourteimour [25] conducted a quasi-experimental study with 70 nursing students, comparing two groups: e-learning with lecture-based teaching (EL + LBT) and e-learning with collaborative learning (EL + CL). Over 14 sessions (each lasting 150 min), self-efficacy was assessed using the College Academic Self-Efficacy Scale. The EL + CL group exhibited significant postintervention improvements in self-efficacy compared with the EL + LBT group (*p* = 0.019). Collectively, these studies demonstrate that higher self-efficacy not only boosts students’ confidence but also motivates them to engage actively with LMS, achieve academic goals, and develop positive perceptions of e-learning platforms.

#### Students’ acceptance of technology

Four studies [23, 26, 28, 36] explored how students’ acceptance of LMS influenced their engagement with e-learning. The findings consistently indicated that students with greater acceptance of LMS were more likely to use these platforms regularly and develop positive perceptions of them.

Chipps et al. [23] found that students who embraced Moodle as a learning tool exhibited increased engagement, frequently accessing the platform for educational materials. Similarly, Mousa et al. [36] reported that while students maintained a positive attitude towards Blackboard, their overall satisfaction with the system was neutral. Marco et al. [28] highlighted barriers to technology acceptance, noting that some students expressed negative attitudes due to limited access to computers. In contrast, Elbasuony et al. [26] reported a statistically significant correlation (*p* = 0.05) between students’ acceptance of LMS and their positive perceptions of e-learning. This emphasised the critical role of acceptance in fostering engagement with digital learning platforms.

### Theme 2: organisational factors

More than half of the included studies (10 out of 19) identified a significant relationship between organisational factors and undergraduate nursing students’ perceptions of LMS [22, 23, 26, 28, 30, 31, 33–36]. Key organisational factors—such as instructors’ influence, access to technology, and the provision of student training and support—were found to shape students’ interactions with LMS. These factors played a pivotal role in influencing students’ engagement, satisfaction, and overall ability to effectively use the platform. The following sections explore how each organisational factor specifically impacts students’ perceptions of LMS.

#### Instructors’ role

Eight studies [23, 24, 26, 28, 31, 33, 34, 37] highlighted the critical role of instructors in shaping students’ perceptions of LMS. Quantitative studies by Shang and Liu [33], Elbasuony et al. [26], Marco et al. [28], and Chipps et al. [23] demonstrated the significant impact of instructors on enhancing student engagement and improving learning outcomes. For example, Shang and Liu [33] reported that 68% of students (*n* = 108) valued instructors’ use of questions and discussions within LMS, which correlated with improved exam performance. Similarly, Elbasuony et al. [26] found that instructors facilitated Blackboard use by creating and managing course content—including lectures, assignments, and evaluations—leading to students’ slightly positive acceptance of the system. Chipps et al. [23] reported that 72.1% of students (*n* = 150) perceived Moodle as user-friendly and beneficial, crediting this perception to instructor support. Marco et al. [28] further demonstrated that instructors’ enthusiasm, expertise, and guidance significantly enhanced student engagement and learning outcomes.

Qualitative findings also underscored the importance of instructor involvement. Shorey et al. [37] reported that instructors’ guidance was essential for first-year students’ satisfaction with LMS. In a mixed-method study, O’Flaherty and Laws [31] revealed that 91% of students noted instructor support with course materials during e-learning improved learning, retention, and perceptions of LMS. Conversely, four studies [23, 24, 26, 30] highlighted that insufficient instructor training and limited technical expertise negatively impacted students’ attitudes towards LMS. These findings underscore the need for adequately preparing instructors to deliver effective e-learning and support students’ successful engagement with LMS.

#### Technology access

Five studies [22–24, 33, 35] underscored the significant impact of technology access on students’ perceptions of LMS. Roudsar et al. [24] reported that students’ satisfaction with e-learning was closely linked to the accessibility of LMS and the availability of learning resources, with 56.3% (*n* = 128) of participants expressing dissatisfaction due to access issues. Similarly, Shang and Liu [33] reported that 84% (*n* = 91) of students had a positive e-learning experience, attributing this to the ease of access provided by the LMS, which enhanced both engagement and satisfaction. Chipps et al. [23] noted that 76.7% (*n* = 160) of participants were able to access Moodle, improving their PEOU and PU of the platform. However, students in rural areas have encountered challenges with Moodle because of slow internet speeds and limited computer resources, leading to dissatisfaction. Renmarker and Carlson [35] reported that access to a web-based platform and associated learning strategies was perceived as positive and supportive of self-directed learning. Similarly, Bloomfield and Jones [22] reported that 78% (*n* = 65) of students evaluated their LMS positively, citing accessibility and the convenience of using it at their preferred times and locations as key factors.

#### Students’ training and support

Five studies [22–24, 33, 35] highlighted the critical role of technology access in shaping students’ perceptions of LMS. Consistent access to LMS platforms and learning resources was strongly associated with improved engagement, satisfaction, and usability.

Roudsar et al. [24] found that students’ satisfaction with e-learning was closely tied to the accessibility of LMS and the availability of learning resources, with 56.3% (*n* = 128) expressing dissatisfaction due to access issues. Conversely, Shang and Liu [33] reported that 84% (*n* = 91) of students experienced positive e-learning outcomes, attributing these to the ease of LMS access, which enhanced engagement and satisfaction.

Chipps et al. [23] observed that 76.7% (*n* = 160) of participants accessed Moodle with ease, which positively influenced their PEOU and PU. However, challenges such as slow internet speeds and limited computer resources negatively impacted students in rural areas, leading to dissatisfaction with Moodle. Renmarker and Carlson [35] noted that access to a web-based platform, combined with effective learning strategies, was perceived as supportive of self-directed learning. Similarly, Bloomfield and Jones [22] reported that 78% (*n* = 65) of students rated their LMS experience positively, emphasising the importance of accessibility and the convenience of using LMS at their preferred times and locations.

### Theme 3: technological factors

Thirteen studies [21–23, 25, 27, 29, 31, 32, 34, 35, 37, 38] highlighted the pivotal role of interactive features within LMS in enhancing student engagement, improving learning outcomes, and fostering positive perceptions of these platforms.

#### LMS features

The importance of LMS features in shaping students’ satisfaction and learning experiences was consistently emphasised across multiple studies. Sáiz-Manzanares et al. [32], in a quasi-experimental post-treatment design with third-year nursing students, reported that 71.9% (*n* = 63) utilised Moodle’s hypermedia resources, including teacher feedback, theoretical materials, video recordings, quizzes, discussion forums, text messages, and automated feedback. These features were found to significantly enhance learning, interaction, outcomes, and overall satisfaction with the LMS.

Coyne et al. [29] noted that nursing students preferred the integration of simulation videos within LMS, highlighting their positive influence on participation and performance. Similarly, Mäenpää et al. [38], in a qualitative study, identified that interactive teaching methods such as video recordings, teacher feedback, and discussions positively shaped students’ attitudes, increasing their engagement, willingness to learn, and enthusiasm.

Chipps et al. [23] found that students rated instructor feedback (72.3%) and discussion boards (73.3%) as the most useful LMS features, which enhanced their PU and acceptance of the system. Bloomfield and Jones [22] reported that 50% of participants identified video clips as the most beneficial feature, significantly improving their learning experiences. Renmarker and Carlson [35] further emphasised the value of interactive features such as PowerPoint presentations, immediate feedback, self-correcting quizzes, animated problem-solving guides, and video simulations, which deepened learning, improved competency, reduced anxiety, and fostered positive e-learning experiences.

Interactive LMS features were also associated with collaboration and communication. Amandu et al. [34], Furnes et al. [27], and Malsakpak and Pourteimour [25] highlighted that discussion boards and diverse teaching methods promoted engagement, facilitated communication, and were particularly beneficial for hesitant students, improving learning outcomes. Moreover, Elbasuony et al. [26], Shorey et al. [37], and Gagnon et al. [21] demonstrated that incorporating interactive materials such as PowerPoint presentations, videos, reflective practices, and discussion forums enhanced participation in online activities and positively influenced learning experiences. O’Flaherty and Laws [31], evaluating an Adobe-connected bioscience module with polling and personalised responses, found that 86% of students valued these features for their final practical test, and 99% reported that e-learning facilitated their achievement of module outcomes.

## Discussion

**Fig. 2:**
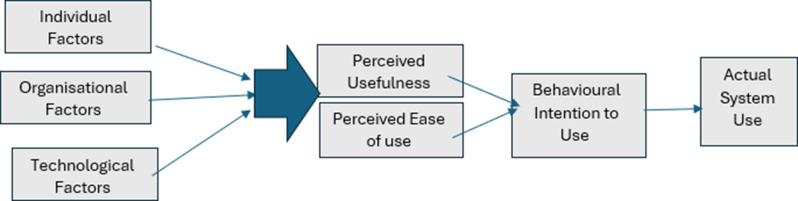
Customised TAM framework for nursing students based on the study results

This systematic review of 19 studies investigated key factors influencing undergraduate nursing students’ perceptions of using LMS. The application of the TAM as a theoretical framework enables an in-depth exploration of PU and PEOU among undergraduate nursing students. TAM provides a structured lens through which to analyse how various factors shape nursing students’ perceptions of LMS use in their academic setting. As illustrated in Fig. 2, three overarching themes emerged from the synthesis: individual factors, organisational factors, and technological factors. These categories encapsulate the key determinants of LMS engagement, providing a structured basis for interpreting the findings.

Previous studies have shown that undergraduate nursing students can be resistant to LMS use [40, 41]. However, this review revealed that, when viewed through TAM, the acceptance and utilisation of LMS among nursing students can significantly influence academic performance, satisfaction, PU, and PEOU. This aligns with previous TAM-based studies on nursing and business students, underscoring the importance of student willingness in actual LMS usage [42, 43].

Notably, this review highlights that PU and PEOU among nursing students appear to be independent of demographic factors such as age and gender, which aligns with recent findings [42]. This contradicts the conclusions of Green [40] and Tarhini et al. [43], who indicated that age and gender significantly influence PU, PEOU, and LMS acceptance. In contrast, our review revealed that factors such as individual, organisational, and technological aspects play a more prominent role, which is consistent with other studies [42, 44].

Individual factors, including digital literacy, prior experience, self-efficacy, and motivation, are critical determinants of nursing students’ interactions with LMS and their overall acceptance of LMS. For example, students with prior technology exposure demonstrated a more positive attitude towards the LMS, positively influencing the PU, PEOU, and overall perceptions of the LMS, as reported in other studies [42, 45, 46]. Self-efficacy has also emerged as a critical factor, aligning with the TAM’s focus on the psychological determinants of technology use [47]. Moreover, self-efficacy interacts with instructor support and feedback, with its impact amplified through constructive instructor feedback [48, 49]. Previous studies have shown that self-efficacy not only affects the PU and PEOU but also increases students’ motivation and confidence in their ability to use LMS effectively [50, 51]. These findings enrich the TAM, highlighting the dynamic relationship between self-efficacy and external factors such as instructor involvement. In particular, the role of instructors significantly influences undergraduate nursing students’ PEOU and PU, a finding that is consistent with prior studies [52]. This review emphasises that sustained motivation, fostered by instructor feedback, support, and guidance, is critical for effective LMS use. Notably, student motivation and training positively affect engagement, knowledge acquisition, and satisfaction with the LMS, which is consistent with prior studies [41, 53, 54].

A strong association was identified between LMS usability, LMS features, and students’ perceptions of and satisfaction with the platform. Nursing students in this review valued LMS features such as immediate feedback, ease of access, video recordings, quizzes, discussion forums, messaging, and storage for theoretical content, all of which influenced PU, PEOU, and LMS acceptance. When students perceive that LMS features support their learning objectives, this encourages LMS use and acceptance. Similarly, previous studies have shown that LMS features such as accessibility, flexibility, assignments, and discussion boards enhance LMS acceptance, engagement, and student satisfaction with the platform [55, 56, 57]. However, some studies have reported that fourth-year undergraduate nursing students face negative experiences with LMS, particularly with features such as interaction, communication, and feedback, primarily due to insufficient training and instructor support [41]. This review confirms that inadequate communication, feedback, and interaction remain significant barriers to LMS use. Therefore, a range of contributing factors influence students’ acceptance and sustained use of LMS [56]. Overall, this review supports the notion that nursing students’ acceptance of LMS is shaped by multiple factors that are essential when LMS are implemented in educational settings [58, 59].

### Implications of the review

This review provides valuable insights into undergraduate nursing students’ acceptance of LMS and the factors acting as facilitators or barriers to effective blended or e-learning. Button, Harrington, and Belan [60] emphasised the importance of e-learning in nursing education, particularly in addressing the growing demand for nursing programmes and the shortage of qualified nursing faculty. Additionally, e-learning equips students with essential lifelong learning skills required to navigate the rapidly evolving global healthcare landscape [61]. The Technology Acceptance Model (TAM) serves as a useful framework for understanding the factors influencing nursing students’ acceptance and perceptions of LMS [62]. A key finding of this review highlights the need to improve digital literacy among both students and instructors to optimise the use of LMS. Educational institutions should consider implementing targeted workshops to address barriers to digital literacy and provide comprehensive training on LMS functionalities. These interventions could enhance students’ perceived ease of use (PEOU) and perceived usefulness (PU) of LMS, thereby fostering greater acceptance and engagement.

The COVID-19 pandemic underscored the importance of LMS in nursing education, as it facilitated the rapid transition to online education. Adapting theoretical and clinical modules to an online format posed significant challenges; however, technological solutions proved instrumental in mitigating these difficulties and demonstrating the value of LMS [63, 64].

Given that most nursing programmes now incorporate LMS, the findings of this review can inform curriculum design by identifying strategies to enhance students’ acceptance and effective use of LMS. These include improving LMS features to align with students’ learning needs, fostering institutional support, and integrating digital literacy training into nursing curricula. Such targeted strategies are essential for ensuring the successful adoption and effective use of LMS in nursing education.

### Recommendations for practice

1. **Support Students**:

- Assess students’ digital literacy upon registration and address gaps through tailored workshops.
- Incorporate LMS orientation sessions to familiarise students with key features and address common challenges.
- Ensure the availability of 24/7 technical support to enhance confidence in LMS use.

2. **Enhance Faculty Skills**:

- Offer regular training to equip faculty with the skills needed to design interactive and engaging LMS modules.
- Highlight the importance of instructor involvement in fostering positive perceptions of LMS.

3. **Improve LMS Design**:

- Simplify LMS interfaces to ensure accessibility and intuitive navigation for nursing students.
- Regularly update LMS features based on feedback from students and instructors.

### Policy recommendations

1. Invest in high-quality LMS platforms that are adaptable to nursing education needs and support interactive learning.
2. Develop policies that promote ongoing faculty and student training in digital literacy and LMS use.
3. Ensure equitable access to digital devices and reliable internet for all students.

### Future research directions

1. Explore the perceptions of postgraduate nursing students and students in other health-related disciplines to broaden the evidence base.
2. Investigate the impact of emerging LMS technologies on student engagement and learning outcomes.
3. Conduct longitudinal studies to examine the sustained impact of LMS interventions on academic performance.

### Strengths and limitations of the review

This review’s systematic approach and reliance on high-quality international primary studies contribute to the robustness of the evidence generated. A notable strength is the application of the TAM, which provided a theoretically grounded framework for synthesising literature on undergraduate nursing students’ perceptions of LMS. However, several limitations should be acknowledged. First, the focus on undergraduate nursing students limits the generalisability of the findings, as perspectives from postgraduate nursing students or those in other health-related disciplines were not included. Future research could explore these populations to provide a more comprehensive understanding of LMS perceptions. Second, the inclusion criteria restricted the review to studies published in English. This may have excluded valuable insights from non-English studies, potentially narrowing the scope of findings. To address this limitation, future studies should consider incorporating non-English literature to capture a broader range of perspectives. Finally, the review’s scope was limited to studies published between 2010 and 2020, with an updated search in 2024. While this ensures the inclusion of recent evidence, it may not fully reflect emerging trends or innovations in LMS use. Expanding the temporal range in future reviews could provide further insights into evolving educational technologies.

## Conclusion

This review offers valuable insights into the factors influencing undergraduate nursing students’ perceptions of LMS. The findings highlight that students’ PU and PEOU are shaped by individual, organisational, and technological factors, each playing a critical role in fostering LMS acceptance within educational contexts. To optimise the effectiveness of LMS, it is essential to prioritise strategies that address these factors. This includes providing targeted digital literacy training, fostering active instructor involvement, and integrating interactive features to enhance engagement. By adopting these approaches, educational institutions can create a more effective and engaging learning environment, ultimately improving nursing students’ experiences and outcomes with LMS.

## Electronic supplementary material

Below is the link to the electronic supplementary material.

Supplementary Material 1

Supplementary Material 2

Supplementary Material 3

## Data Availability

No datasets were generated or analysed during the current study.

## Abbreviations

LMS: Learning Management system
Moodle: Modular-object- Oriented Dynamic Learning Environment
F2F: Face to Face
PU: Perceived usefulness
PEOU: Perceived ease of use
BL: Blended Learning
TAM: Technology acceptance model
CAT: Critical appraisal tool
RCT: Randomised controlled trial
CASP: Critical Appraisal Skills Programme
MKPCT: Milton Keynes Primary Care Trust
MMAT: Mixed Methods Appraisal Tool
JBI: Joanna Briggs Institute
CINAHL: Cumulative Index to Nursing and Allied Health Literature
MEDLINE: Medical Literature Analysis and Retrieval System Online
MeSH: Medical Subject Headings
PRISMA: Preferred Reporting Items for Systematic Reviews and Meta-Analyses
EMBASE: Excerpta Medica database
