# Supplementary material for: Key factors influencing undergraduate nursing students’ perceptions of the use of learning management systems: a systematic literature review
Source: BMC Nurs. 2025 Mar 26;24:323. doi: 10.1186/s12912-025-02962-9 (PMC11948785; doi:10.1186/s12912-025-02962-9)
Supplement: Supplementary file 1 — Supplementary Material 1 [file 12912_2025_2962_MOESM1_ESM.docx]

**Supplementary file 1.**

**Table1. Search terms and their synonyms**

| Search terms | Learning management system | Nurs* | Experience | Student | Blended learning |
| --- | --- | --- | --- | --- | --- |
| Synonyms | Platform | Nurses/nursing/nurse | Perception  Perspective  View  Evaluation | Undergraduate  Learner  Bachelor | e-learning |

**Table2**. **Combination of the search terms with Boolean logic commands**

| Learning management system AND nurs* AND students AND perception | Learning management system AND nurs* AND undergraduate OR bachelor students AND experience | Platform AND blended learning AND nurs* AND student AND perception | Blended learning AND nurs* AND student OR undergraduate AND view | Blended learning AND e-learning AND nurs* AND students OR learner AND perspective | e-learning AND platform AND nurs* AND undergraduate AND experience OR evaluation |
| --- | --- | --- | --- | --- | --- |

**Table 3. Initial results of the literature search of five databases**.

| **Database** | **Initial result** | **Database** | **Initial result** |
| --- | --- | --- | --- |
| EMBASE | 29 | Cochrane Library | 116 |
| CINHAL Plus | 38 | Reference lists | 3 |
| Medline | 156 |  |  |
| Web of Science | 195 |  |  |

**Table 4. Reasons of full text exclusion**

| 6Excluded | Title | Authors | Year | Reseaon |  |
| --- | --- | --- | --- | --- | --- |
| 1 | First year undergraduate nursing students’ perceptions of the effectiveness of blended learning approaches for nursing numeracy | O'Reilly et al | 2020 | the study didn't address blended learning from the concept of a learning management system |  |
| 2 | \Perception of Nursing Students regarding Blended Learning Method at The University of Lahore, Pakistan | Afzal,M.,Ali,T. and Gilani,S. | 2019 | study didn't address learning via Learning management system |  |
| 3 | The Effect of Blended Learning on Nursing Students’ Knowledge | Eka,N.,Houghty,G. and Juniarta | 2019 | focus of the study was on the knowledge of the students not on LMS |  |
| 4 | Evaluation of E-learning program versus traditional lecture instruction for undergraduate nursing students in a faculty of nursing | Abdelaziz et al | 2011 | study didn't focus on Learning management system |  |
| 5 | Effect of Blended Learning on Newly Nursing Student's Outcomes Regarding New Trends in Nursing Subject at Ain Shams University | Mersal,F. and Mersal,N. | 2014 | study didn't focus on Learning management system |  |
| 6 | Determining attitudes toward e‑learning: what are the attitudes of health professional students? | Ayla Güllü1  · Mustafa Kara2  · Şenay Akgün | 2022 | Study didn't focus on Learning management system |  |
| 7 | Efficacy of a blended learning programme in enhancing the communication skill competence and self-efficacy of nursing students in conducting clinical handovers: a randomised controlled trial | Jessie Yuk Seng Chung1 , William Ho Cheung Li2*, Ankie Tan Cheung2 , Laurie Long Kwan Ho2 and Joyce Oi Kwan Chung | 2022 | Study didn't focus on Learning management system |  |
| 8 | Effectiveness and learning experience from undergraduate nursing students in surgical nursing skills course: a quasiexperimental study about blended learning | Yan Ran Li1 , Zong Hao Zhang1 , Wen Li1 , Pan Wang1 , Shu Wen Li1* , Dan Su1 and Ting Zhang1 | 2023 | Didn’t focus on LMS |  |
| 9 | Cognitive and Non-Cognitive Determinants of Academic Performance in Nursing | Josephine N. Amadi,Regidor Poblete,Glory B. Obong,Chijike Canis Irodi,Nonye Celestina Irodi | 2023 | Didnt address LMS |  |
| 10 | Effect of blended self-directed learning on nursing students: Quasi-experimental approach | Govindan SN, Singh HKD, Ling LW, Sekar M. | 2023 | Study focus on self directed learning not LMS. |  |
| 11 | Nursing students’ knowledge, attitude, self efficacy in blended learning of cardiopulmonary resuscitation: a randomized controlled trial | Moon,H. and Hyun,H. | 2019 | Study focus on the education of CPR not one the delivery of online learning |  |
