# Supplementary material for: Key factors influencing undergraduate nursing students’ perceptions of the use of learning management systems: a systematic literature review
Source: BMC Nurs. 2025 Mar 26;24:323. doi: 10.1186/s12912-025-02962-9 (PMC11948785; doi:10.1186/s12912-025-02962-9)
Supplement: Supplementary file 2 — Supplementary Material 2 [file 12912_2025_2962_MOESM2_ESM.docx]

###### Supplementary File 2.

###### Data Extraction Table

| **Author, Year & Place of Publication, Journal Name** | **Study Aim** | **Study Design** | **Outcome Measures/ Instruments** | **sample Size** | **Name of LMS** | **Key findings** | **Quality review** | **conclusion** | **Limitation** |  |
| --- | --- | --- | --- | --- | --- | --- | --- | --- | --- | --- |
|  |  |  |  |  |  |  |  |  |  |  |
| CHIPPS, J., KERR, J., BRYSIEWICZ, P. and WALTERS, F.,( 2015). A Survey of University Students’ Perceptions of Learning Management Systems in a Low-Resource Setting Using a Technology Acceptance Model. CIN: Computers, Informatics, Nursing, 33(2), pp.71-77. **South Africa** | to assess various factors affecting students perceptions while using LMS | Descriptive Survey | Questionnaires, Open-ended questions utilising four-point Likert scale calculation done with  alpha of Cronbach | total of 213 nursing students, no sampling technique done, 69.5% female and30.5 %male. | Moodle | perceived ease of use LMS, features of LMS perceived usefulness. Factors including individual, organisation and LMS influence students’ perceptions | 19/22 using MKPCT | students perceived ease of use and usefulness of LMS if they had computer literacy. Train students' and orient them on using LMS with regular support and follow up from organisation | deficit in variables of tracking rural students' |  |
| Elbasuony, M., Gangadharan, P. and R., J., (2018). Undergraduate Nursing Students’ Perception and Usage of E-Learning and Blackboard Learning System. Middle East Journal of Nursing, 12(2), pp.3-13. **Saudi Arabia** | To investigate undergraduate nursing students perceived of knowledge, attitude and the use of Blackboard LMS | Descriptive | computrised questionnaires/ analysis of data done with test of correlation (r). the association between demographic data were explored with (χ2) test of chi square. | 80 female students of undergraduate nursing /Convenient sampling. | Black Board | (72%) had perception of knowledge on LMS and 64% of students’ had level of average skills on the basic computer and 40 % showed good at Blackboard applications | 19/22 using MKPCT | students' perceived high level of knowledge and attitude toward Black board LMS. | All participants were female, low proportion of the participants were included in the study; it was carried on a single institution |  |
| Roudsar, D., Haghani, H., Dehnad, A., Ghalesari, M., Shoub, M. and Rokni, M., (2019). Investigation of students' academic participation in combined learning based on learning management system. Medicni perspektivi (Medical perspectives), 24(4), pp.12-18. **Iran** | To explore the association between students' academic participation on LMS and the degree of their academic participation. | a descriptive cross-sectional study | satisfaction of students' on LMS / a questionnaire on a scale of  Fredericks, Blumenfeld and Paris of students’ academic participation, questionnaires of 2005 analysed with Cronbach's alpha 0.66 | undergraduate nursing students 229 /sampling method based on entry criteria / 52.8 female | not stated | 75.5% of students were familiar with LMS, (56.3%) had a problem with access of LMS. Findings showed there was no association between students' participation and other variables | 20/22 using MKPCT | workshops and training is recommended to support students’ academic participation and faculties | no limitation had been reported in the study |  |

| **Author, Year & Place of Publication, Journal Name** | **Study Aim** | **Study Design** | **Outcome Measures/ Instruments** | **sample Size** | **Name of LMS** | **Key findings** | **Quality review** | **conclusion** | **Limitation** |  |
| --- | --- | --- | --- | --- | --- | --- | --- | --- | --- | --- |
|  |  |  |  |  |  |  |  |  |  |  |
| Coyne, E., Frommolt, V., Rands, H., Kain, V. and Mitchell, M., 2018. Simulation videos presented in a blended learning platform to improve Australian nursing students' knowledge of family assessment. Nurse Education Today, 66, pp.96-102. **Australia (2)** | to assess student’s knowledge and confidence on self-assessment after watching simulation videos on family assessment in a module of child and health | exploratory descriptive survey | engagement of students was evaluated by statistician of the university using data tacked through website as a LMS measuring time, duration and number of students' access the platform | 619 students' who enrolled in the course from three campuses, responding were 163 and 90% were female | not stated | Students' perceived positively in terms of knowledge of family assessment attained via simulation videos. Positive an association between engagement of students on LMS and academic accomplishments. two factors influencing students were budget and motivation | 19//22 using MKPCT | student preferred the interactive learning mode, recommend more BL model for other nursing subjects | low of participants’ rate, biased may induced for students who were motivated more. |  |
| Meedya, S., Moroney, T., Nielsen, W. and Najafi Bokat, I., (2019). Digital explanations and nursing students’ perception of learning science. Nurse Education in Practice, 41, p.102636, **Australia(3)** | to assess experience of nursing students via assessment of formative in learning science using digital explanation | Sequential explanatory mixed method | self-reported survey and interview in anatomy and physiology module to measure students' perceptions. Analysis done with SPSS | 428 students of 1st year from five different colleges /86% were female / less than 26 years | not stated | Students feedback was positive, students attain knowledge, had fun and useful experience in science topics | 31/34 using MMAT | students generating digital videos had facilitate their learning in clinical settings, good to adopt policy of introducing digital in teaching nursing, students' motivation is important | the students’ digital assignments were not graded which induced lack of motivation and there was no opportunity to verify findings with participants |  |
| Marco, L., Venot, A. and Gillois, P., (2017). Does the acceptance of hybrid learn affect learning approaches in France? Journal of Educational Evaluation for Health Professions, 14, p.24. **France** | To assess the effect of acceptance of a hybrid learning style on students' learning | Survey | students learning approaches affected by LMS. Survey using questionnaire to assess students' learning attitude toward LMS/Spearman coefficient (rs) | 38 students of midwifery | not stated | there was an association between positive acceptance of model and deep learning. Students developed skills of time management | 18/22 using MKPCT | motivation is important to enhance acceptance and intention to use hybrid learning, students complained with easy distract while using LMS and organisation of study time. | rate of responses was high compared to small sample |  |

| **Author, Year & Place of Publication, Journal Name** | **Study Aim** | **Study Design** | **Outcome Measures/ Instruments** | **sample Size** | **Name of LMS** | **Key findings** | **Quality review** | **conclusion** | **Limitation** |  |
| --- | --- | --- | --- | --- | --- | --- | --- | --- | --- | --- |
|  |  |  |  |  |  |  |  |  |  |  |
| O'Flaherty, J. and Laws, T., (2014). Nursing Student's Evaluation of a Virtual Classroom Experience in Support of Their Learning Bioscience. Journal of Nurse Education in Practice,14, p654-659. **Australia (1)** | to explore students' experience of virtual classrooms using a comparison between face to face and virtual learning in anatomy and physiology | Mixed Method | student’s satisfaction, final grades and experience was assessed, in two groups using Course Evaluation Instrument (CEI), survey questionnaire  as well as free text responses | 236 of 1st year students in physiology and anatomy | Adobe Connect Software | 86% of students perceived virtual learning as valuable aid for practical test and enhance their participation in virtual | 29/34 using MMAT | Valuable experience which helped students to attain knowledge and interaction with others | . no limitation had been reported in the stud |  |
| Shang, F. and Liu, C., (2018).  Blended learning in medical physiology improves nursing students’ study efficiency. Advances in Physiology Education, 42(4), pp.711-717. **China** | to assess skills and knowledge of students’ attained via blended learning | survey | Students opinion and feedback on blended learning in a module of physiology, data analysis done with SPSS and T-test. | students divided into three sections, each section with 54 or 55. | platform of Tsinghua Education Online (THEOL) | students' performance was significantly improved in the final exam; students' feedback was positive. Students preferred blended learning over F2E due to flexibility and development of independency skills. | 19/22 using MKPCT | more interactive blended courses are needed to improve students' outcomes. regular support and feedback were needed for students to facilitate learning. Lack of socialisation in blended learning were reported by students | no limitation had been reported in the study |  |
| Sáiz-Manzanares, M., Escolar-Llamazares, M. and Arnaiz González, Á., (2020). Effectiveness of Blended Learning in Nursing Education. International Journal of Environmental Research and Public Health, 17(5), p.1589. **Spain** | to assess students results using LMS | Quasi-experimental post treatmen | comparison of two groups (experimental group with blended. and control group). Scales of by Román & Poggiali using, SPSS, ANOVA and the (ACRAr) | 120 3rd students/ more female students using convenience sampling | Moodle LMS | students on two groups scored good results but students at BL group got high marks, students appreciate the accessibility and availability of content in LMS which was very useful and helped them to acquire knowledge and skills. | 8/9 using JBI | . BL was very effective in enhancing students' learning, LMS could be used as analytical tool which helped instructors to detect students with weak performance | single settings, specific knowledge and specific study designs |  |

| **Author, Year & Place of Publication, Journal Name** | **Study Aim** | **Study Design** | **Outcome Measures/ Instruments** | **sample Size** | **Name of LMS** | **Key findings** | **Quality review** | **conclusion** | **Limitation** |  |
| --- | --- | --- | --- | --- | --- | --- | --- | --- | --- | --- |
|  |  |  |  |  |  |  |  |  |  |  |
| Furnes, M., Kvaal, K. and Høye, S., (2018). Communication in mental health nursing - Bachelor Students' appraisal of a blended learning training programme - an exploratory study. BMC Nursing, 17(1). **Norway**. | To outline the students' evaluation of blended learning in communication skills in module of mental health nursing | Mixed Method exploratory design | The questionnaire was used to measure students’ perceptions via learning outcomes. Analysis was done through SPSS and The Kruskal Wallis test | 169 of 3rd year students | not stated | students' reported improvement of their knowledge, skills and satisfaction. They perceived BL as valuable and important for practice as well | 31/34 using MMAT | students tend to learn more via role play. The students recommended the role of teachers in role play. Early involvement and participation of students while creating the learning modules would be recommended | researchers develop the tool of questionnaire at the University. Some participants returned to questionnaire   weeks after the module. Risk of bias may induce by involvement of the teacher course during evaluation |  |
| Gagnon, M., Gagnon, J., Desmartis, M. and Njoya, M., 2013. The Impact of Blended Teaching on Knowledge, Satisfaction, and Self-Directed Learning in Nursing Undergraduates: A Randomized, Controlled Trial. Nursing Education Perspectives, 34(6), pp.377-382. **Canada** | to explore the effects of BL intervention via Internet-based tutorials with some F2F lectures in a module of introduction to research | Randomised Control Trials | measuring 3 learning outcome knowledge, satisfaction and self-directed learning using midterm and finals, questionnaire) and scale of fisher's result analysis done via covariance ANCOVA | 120 of 1st year students of undergraduate nursing /more female students. | not stated | Although there was no direct influence on students' knowledge  , satisfaction and SDLR, BL had an impact on students' motivation | Low to moderate Biased with CASP tool | BL using internet based comparable to F2F teaching, BL is better suits some students depending on degree of motivation | blinding of students was not maintained, various schedule between two arms may impact students motivation, and results, students in two arms received different attention |  |
| Bloomfield, J. and Jones, A., (2013). Using E-Learning to Support Clinical Skills Acquisition: Exploring The Experiences and Perceptions of Graduate First-Year Pre-Registration Nursing Students — A Mixed Method Study. Nurse Education Today,33(), pp.1605-1611. **U.K** | to investigate the perceptions of 1st yr students on e-learning in learning clinical skills . | A mixed method | students' perceptions and experience while using e-learning for clinical skills. descriptive statistics to analysis the data. | 83 cohort group and focus group 15 students of 1st yr. students'/ 76% were female students | not stated | students' perceived e-learning positively and valuable experience to acquire practical skills. e-learning was a Flexible in time and place, ease to access and facilitate autonomy of students. students preferred the visual aids was video to attain clinical practice | 30/34 using MMAT | Flexibility to access the materials at any time and place was useful to introduce in accelerated nursing program to fulfil students’ demands. Quality of e-learning system, accessibility and simulating resources are important to enhance student experiences, knowledge and clinical skills. | Sample size was small, the participants were from motivated students’ who had their view and preferences toward e-learning which in turn may affect results |  |

| **Author, Year & Place of Publication, Journal Name** | **Study Aim** | **Study Design** | **Outcome Measures/ Instruments** | **sample Size** | **Name of LMS** | **Key findings** | **Quality review** | **conclusion** | **Limitation** |  |
| --- | --- | --- | --- | --- | --- | --- | --- | --- | --- | --- |
|  |  |  |  |  |  |  |  |  |  |  |
| Mäenpää, K., Järvenoja, H., Peltonen, J. and Pyhältö, K., (2020). Nursing students’ motivation regulation strategies in blended learning: A qualitative study. Nursing & Health Sciences, pp.1-10. **Finland.** | to obtain deep insight of motivation regulation strategies on nursing students  (MR) and the factors influencing their use of LMS throughout three years of nursing | Qualitative | deductive, retrospective recalling data was collected through interview and analysis was done to outline students' experiences. | 12 students of 3rd year / 7 female 58 % and 5 male 42%/ purposive sampling | not stated | 7 motivational strategies were identified including structuring the environment,  consequate of self, oriented goal self-talk, efficacy, regulation of emotion, value regulation, and enhancement of interest . Factors like Individual and situational had an impact students' LMS usage and sustainability. | 16/20 using CASP | improved knowledge on MR which influencing nursing students, and how to increase MR in BL nursing modules. Findings could be useful for instructors to facilitate MR while learning | sample size was small, single place, culture and institution factors may have influence on the study results |  |
| Shorey, S., Siew, A. and Ang, E., (2018). Experiences of nursing undergraduates on a redesigned blended communication module: A descriptive qualitative study. Nurse Education Today, 61, pp.77-82. **Singapore** | To investigate experience of 1st year- nursing students on blended learning in a module of communication | Qualitative | Exercise of online reflective was given to students on their final day of the class and thematic analysis was followed for data analysis | 74 1st year students/ 85.1% were female and 14.9% were male /sampling not stated. | not stated | 6 themes were identified with subthemes. 6 themes were: useful experience of BL, online activities were valuable, assessments were meaningful, enhancement of education of inter-professional, enrichment of personal experience and positive experience and helpful feedback. | 16/20 using CASP | BL styles is useful to use in module of communication for nursing students. It boosts confidence of students and enhance students’ knowledge and skills. Appreciation of inter-professional learning while teaching nursing students | interview was not face to face, single settings and one cohort of students which would impact generalisation of the study outcomes |  |

| **Author, Year & Place of Publication, Journal Name** | **Study Aim** | **Study Design** | **Outcome Measures/ Instruments** | **sample Size** | **Name of LMS** | **Key findings** | **Quality review** | **conclusion** | **Limitation** |  |
| --- | --- | --- | --- | --- | --- | --- | --- | --- | --- | --- |
|  |  |  |  |  |  |  |  |  |  |  |
| Renmarker, E. and Carlson, E., (2019). Evaluation of Swedish nursing students’ experience of a web-based platform for drug calculation. Nurse Education in Practice, 38, pp.89-95. **Sweden** | To assess the experience of nursing students and their use of web-based platform for drug calculation | Evaluation study | a comparison of students’ experience on semester one and six. SPSS was used for data analysis. Data description was done with via mean, median and descriptive analysis | 95 of nursing students /convenience sampling/F were 80%, M were 10.5 other gender 2.1 | web based platform learning platform | most of students had positive learning experience on platform  students perceived ease of use of platform (p≤0.040)  (p≤0.050) perceived positive experience in semester six compared to semester one | 18/22 using MKPCT | Students reported a Useful, supported and benefit learning experience of drug calculation in the platform. | findings of the study difficult to generalised as there was a small number of participants and single settings |  |
| Amandu, G., Muliira, J. and Fronda, D., (2013). Using Moodle E-learning Platform to Foster Student Self-directed Learning: Experiences with Utilization of the Software in Undergraduate Nursing Courses in a Middle Eastern University. Procedia - Social and Behavioral Sciences, 93, pp.677-683. **Oman** | To explore successful impact of Moodle LMS in promoting SDL in undergraduate nursing students' | Observational study | evaluating experience of students on Moodle LMS in 3 various nursing modules | undergraduate nursing Students enrol in module of health assessment, nursing administration and health promotion. Sample size and technique not specified. | Moodle LMS | There were many Benefits attained by students using Moodle e.g. attainment of knowledge and skills, flexibility of the module, enhancement of students’ engagement and participation, good attitude was adopted, improve SDL, facilitate students’ motivation and organisation of time schedule | 5/6 using JBI | Learning via LMS improves students’ achievements, collaboration, self- awareness, and life-long learning experience | no sampling technique and sample size was not specified. Using single settings. |  |
| Yang, Y. and Lin, N., (2010). Internet perceptions, online participation and language learning in Moodle forums: A case study on nursing students in Taiwan. Procedia - Social and Behavioral Sciences, 2(2), pp.2647-2651. **Taiwan** | To explore relationship of students' perception using internet and their participation on writing skills using Moodle LMS for nursing students in English module | Case study | students’ perception and participations on Moodle LMS using questionnaires, Alpha co-efficient with 0.87 and 0.91 | Junior students 47/sampling not stated | Moodle LMS | Students’ appreciate the usefulness of LMS, LMS enhanced students’ confidence in using LMS independently, promote students’ self-efficacy | 18/20 using CASP | Students' Positive attitude toward LMS influenced them to participate more in LMS. | not stated |  |

| **Author, Year & Place of Publication, Journal Name** | **Study Aim** | **Study Design** | **Outcome Measures/ Instruments** | **sample Size** | **Name of LMS** | **Key findings** | **Quality review** | **conclusion** | **Limitation** |  |
| --- | --- | --- | --- | --- | --- | --- | --- | --- | --- | --- |
|  |  |  |  |  |  |  |  |  |  |  |
| Hemmati Malsakpak M, Pourteimour S. (2024).Comparison of the Effects of E-learning Blended with Collaborative Learning and Lecture-Based Teaching Approaches on Academic Self-Efficacy among Undergraduate Nursing Students: A Quasi-Experimental Study. J Adv Med Educ Prof. 2024;12(2):102-110. DOI: 10.30476/JAMP.2024.99100.1828.  Iran | To compare the effects of blended learning (E-learning combined with Collaborative Learning) and Lecture-Based Teaching on academic self-efficacy among undergraduate nursing students. | Quasi-experimental pre-/post-test design with two comparison intervention groups. | Academic self-efficacy was measured using the College Academic Self-Efficacy Scale (CASES). Demographic information form. | 70 undergraduate nursing students female and male. | Not specified | No significant difference in CASES scores between students taught using E-learning combined with Lecture-Based Teaching (EL+LBT) and those using E-learning combined with Collaborative Learning (EL+CL) before interventions.  After interventions, the CL+EL approach resulted in higher increases in CASES scores compared to the EL+LBT approach.  Female students in the CL+EL group showed significantly higher self-efficacy scores compared to male students | 8/9 using JBI | Integrating E-learning with Collaborative Learning significantly enhances academic self-efficacy among undergraduate nursing students compared to Lecture-Based Teaching combined with E-learning. This approach is particularly effective in boosting female students' self-efficacy | the lack of a control group and potential external factors influencing academic performance were not considered. |  |

| **Author, Year & Place of Publication, Journal Name** | **Study Aim** | **Study Design** | **Outcome Measures/ Instruments** | **sample Size** | **Name of LMS** | **Key findings** | **Quality review** | **conclusion** | **Limitation** |  |
| --- | --- | --- | --- | --- | --- | --- | --- | --- | --- | --- |
|  |  |  |  |  |  |  |  |  |  |  |
| Mousa KM, Elsawy MM, Abd Elkodoos RF. (2022). Attitude and Satisfaction of Undergraduate Nursing Students in Cairo University Toward Usage of Blackboard Learning Management System. Assiut Sci Nurs J. 2022;10(28):102-110. doi: 10.21608/ASNJ.2022.[36][0551.1317. Egypt | To assess the attitude and satisfaction of undergraduate nursing students in Cairo University toward usage of Blackboard LMS. | Exploratory Cross-sectional design | Blackboard electronic questionnaire with three parts: demographic data, perceived attitude, and student satisfaction scale | 1000 undergraduate nursing students  40.5% of the participants were male, and 59.5% were female. | BALCK BOARD LMS | Positive attitude and neutral satisfaction among students towards Blackboard LMS Significant impact on teaching and learning outcomes Recommendations for more training programs and further studies in different settings | 22/22  using  MKPCT | . Positive attitude and neutral satisfaction towards Blackboard LMS. Emphasizes the role of students' attitudes and satisfaction in teaching and learning outcomes | Did not account for potential external factors influencing academic performance |  |
