# Supplementary material for: Key factors influencing undergraduate nursing students’ perceptions of the use of learning management systems: a systematic literature review
Source: BMC Nurs. 2025 Mar 26;24:323. doi: 10.1186/s12912-025-02962-9 (PMC11948785; doi:10.1186/s12912-025-02962-9)
Supplement: Supplementary file 3 — Supplementary Material 3 [file 12912_2025_2962_MOESM3_ESM.docx]

###### Supplementary File3.

###### Critical Appraisal of the Included Studies

###### A. CASP Appraisal criteria (Randomised control trial)

| **study details**  Gagnon, M., Gagnon, J., Desmartis, M. and Njoya, M., (2013). The Impact of Blended Teaching on Knowledge, Satisfaction, and Self-Directed Learning in Nursing Undergraduates: A Randomized, Controlled Trial. Nursing Education Perspectives, 34(6), pp.377-382. Canada | |
| --- | --- |
| (A) Are the result of the study Valid | Yes, Can't tell , No |
| 1 .Did the trial address a clearly focused issue? | Yes |
| 2.Was the assignment of patients to treatments randomised | yes |
| 3.Were patients, health workers and study personnel blinded | No |
| 4.Were the groups similar at the start of the trial? | Yes |
| A.5.Aside from the experimental intervention, were the groups treated equally | No |
| 6. Were all of the patients who entered the trial properly accounted for at its conclusion? | Yes |
| (B) What are the results? | Yes, Can't tell , No |
| 7. How large was the treatment effect? | no significant difference between two groups in satisfaction and knowledge. No difference between arms in SDLR score but after adjusting for motivation as covariate a significant difference on the SDLR score was found in post intervention |
| 8.How precise was the estimate of the treatment effect? | p=0.0005significant positive effect on satisfaction was motivation p=.0005 for both groups |
| (C) Will the results help locally? | Yes, Can't tell , No |
| 9.9. Can the results be applied in your context? (or to the local population?) | Yes |
| 10.Were all clinically important outcomes considered? | Yes |
| 11. Are the benefits worth the harms and costs? | Yes |

Quality of this paper was low to moderate risk of bias because the location was not concealed

###### B. CASP Appraisal criteria (Qualitative studies)

| **Study detail** | Mäenpää, K., Järvenoja, H., Peltonen, J. and Pyhältö, K., (2020). Nursing students’ motivation regulation strategies in blended learning: A qualitative study. Nursing & Health Sciences, pp.1-10. | Shorey, S., Siew, A. and Ang, E., (2018). Experiences of nursing undergraduates on a redesigned blended communication module: A descriptive qualitative study. Nurse Education Today, 61, pp.77-82. |
| --- | --- | --- |
| **Critical appraisal of CASP**  **(Qualitative)** | **Yes (2) Can't tell (1) No (0)** | **Yes (2) Can't tell (1) No (0)** |
| 1.Was there a clear statement of the aims of the research? | 2 | 2 |
| 2.Is a qualitative methodology appropriate? | 2 | 2 |
| 3. Was the research design appropriate to address the aims of the research? | 2 | 2 |
| 4. Was the recruitment strategy appropriate to the aims of the research? | 1 | 1 |
| 5. Was the data collected in a way that addressed the research issue? | 2 | 1 |
| 6. Has the relationship between researcher and participants been adequately considered? | 1 | 2 |
| 7. Have ethical issues been taken into consideration? | 1 | 2 |
| 8. Was the data analysis sufficiently rigorous? | 1 | 2 |
| 9. Is there a clear statement of findings? | 1 | 2 |
| 10. How valuable is the research? | 2 | 2 |
| **Total score** | **18/20** | **18/20** |

Rating=18-20 good in quality,15-17 moderate in quality, 14 and less= low in quality.

###### C. CASP Appraisal criteria (Case study)

| **Study detail**  Yang, Y. and Lin, N., (2010). Internet perceptions, online participation and language learning in Moodle forums: A case study on nursing students in Taiwan. Procedia - Social and Behavioral Sciences, 2(2), pp.2647-2651. Taiwan | |
| --- | --- |
| **(A) Are the result of the study Valid** | **Yes (2) Can't tell (1) No(0)** |
| 1. Did the study address a clearly focused issue? | 2 |
| 2.Did the authors use an appropriate method to answer their question? | 2 |
| 3. Were the cases recruited in an acceptable way? | 1 |
| 4. Were the controls selected in an acceptable way? | 2 |
| 5. Was the exposure accurately measured to minimise bias? | 1 |
| 6.(a) Aside from the experimental intervention, were the groups treated equally? | 1 |
| 6. (b) Have the authors taken account of the potential confounding factors in the design and/or in their analysis? | 2 |
| **Section B: What are the results?** | **Yes Can't tell No** |
| 7. How large was the treatment effect? | Significant (2) |
| 8. How precise was the estimate of the treatment effect? | .08-0.09 (2) |
| 9. Do you believe the results? | 2 |
| **Section C: Will the results help locally?** | **Yes Can't tell No** |
| 10. Can the results be applied to the local population? | 2 |
| 11. Do the results of this study fit with other available evidence? | 2 |
| **Total score** | **19/22** |

###### D. MKPCT Appraisal criteria (Surveys).

| **Yes(2),Can’t tell(1),No (0)** | **CHIPPS, J., KERR, J., BRYSIEWICZ, P. and WALTERS, F.,( 2015). A Survey of University Students’ Perceptions of Learning Management Systems in a Low-Resource Setting Using a Technology Acceptance Model. CIN: Computers, Informatics, Nursing, 33(2), pp.71-77.**  **South Africa** | **Coyne, E., Frommolt, V., Rands, H., Kain, V. and Mitchell, M., (2018). Simulation videos presented in a blended learning platform to improve Australian nursing students' knowledge of family assessment. Nurse Education Today, 66, pp.96-102. Australia** | **Elbasuony, M., Gangadharan, P. and R., J., (2018). Undergraduate Nursing Students’ Perception and Usage of E-Learning and Blackboard Learning System. Middle East Journal of Nursing, 12(2), pp.3-13. Saudi Arabia** | **Shang, F. and Liu, C., (2018). Blended learning in medical physiology improves nursing students’ study efficiency. Advances in Physiology Education, 42(4), pp.711-717. China** | **Marco, L., Venot, A. and Gillois, P., (2017). Does the acceptance of hybrid learning affect learning approaches in France? Journal of Educational Evaluation for Health Professions, 14, p.24. France** |
| --- | --- | --- | --- | --- | --- |
| 1. Did the study address a clearly focused issue**?** | 2 | 2 | 2 | 2 | 2 |
| 2. Did the authors use an appropriate method to answer their question? | 2 | 2 | 2 | 2 | 2 |
| 3. Were the subjects recruited in an acceptable way? | 1 | 1 | 1 | 1 | 1 |
| 4. Were the measures accurately measured to reduce bias? | 1 | 1 | 1 | 1 | 1 |
| 5.Were the data collected in a way that Addressed the research issue? | 2 | 2 | 2 | 2 | 2 |
| 6. Did the study have enough participants to Minimize the play of chance? | 1 | 1 | 1 | 2 | 1 |
| 7.How the results presented and what is the main result? | 2 | 2 | 2 | 2 | 2 |
| 8.Was the data analysis sufficiently rigorous? | 2 | 2 | 2 | 2 | 2 |
| 9.Is there a clear statement of findings? | 2 | 2 | 2 | 2 | 2 |
| 10.Can the results be applied to the local population? | 2 | 2 | 2 | 2 | 2 |
| 11.How valuable is the research? write comments here | 2 | 2 | 2 | 1 | 1 |
| **Total Score** | **19/22** | **19/22** | **19/22** | **19/22** | **18/22** |

###### E. MKPCT Appraisal criteria (Cross-sectional studies)

| **Yes (2)/ can't tell (1) /No(0)** | **Roudsar, D., Haghani, H., Dehnad, A., Ghalesari, M., Shoub, M. and Rokni, M.,( 2019). Investigation of students' academic participation in combined learning based on learning management system. Medicni perspektivi (Medical perspectives), 24(4), pp.12-18. Iran** | **Renmarker, E. and Carlson, E., (2019). Evaluation of Swedish nursing students’ experience of a web-based platform for drug calculation. Nurse Education in Practice, 38, pp.89-95. Sweden** | **Mousa KM, Elsawy MM, Abd Elkodoos RF. Attitude and Satisfaction of Undergraduate Nursing Students in Cairo University Toward Usage of Blackboard Learning Management System. Assiut Sci Nurs J. 2022;10(28):102-110. doi: 10.21608/ASNJ.2022.120551.1317. Egypt** |  |  |
| --- | --- | --- | --- | --- | --- |
| 1. Did the study address a clearly focused issue**?** | 2 | **2** | **2** |  |  |
| 2. Did the authors use an appropriate method to answer their question? | 2 | **2** | **2** |  |  |
| 3. Were the subjects recruited in an acceptable way? | 1 | **1** | **2** |  |  |
| 4. Were the measures accurately measured to reduce bias? | 1 | **1** | **2** |  |  |
| 5.Were the data collected in a way that Addressed the research issue? | 2 | **2** | **2** |  |  |
| 6. Did the study have enough participants to Minimize the play of chance? | 2 | **2** | **2** |  |  |
| 7.How the results presented and what is the main result? | 2 | **2** | **2** |  |  |
| 8.Was the data analysis sufficiently rigorous? | 2 | **1** | **2** |  |  |
| 9.Is there a clear statement of findings? | 2 | **1** | **2** |  |  |
| 10.Can the results be applied to the local population? | 2 | **2** | **2** |  |  |
| 11.How valuable is the research? write comments here | 2 | **2** | **2** |  |  |
| **Total Score** | **20/22** | **18/22** | **22/22** |  |  |

###### F. MMAT appraisal criteria (Mixed-Method designs)

| **Yes (2), Can't tell(1), No (0)** | Meedya, S., Moroney, T., Nielsen, W. and Najafi Bokat, I.,( 2019). Digital explanations and nursing students’ perception of learning science. Nurse Education in Practice, 41, p.102636 | Paper 2 O'Flaherty, J. and Laws, T., (2014). Nursing Student's Evaluation of a Virtual Classroom Experience in Support of Their Learning Bioscience. Journal of Nurse Education in Practice,14, p654-659. **Australia** | Furnes, M., Kvaal, K. and Høye, S., (2018). Communication in mental health nursing - Bachelor Students' appraisal of a blended learning training programme - an exploratory study. BMC Nursing, 17(1).Norway. | Bloomfield, J. and Jones, A., (2013). Using E-Learning to Support Clinical Skills Acquisition: Exploring The Experiences and Perceptions of Graduate First-Year Pre-Registration Nursing Students — A Mixed Method Study. Nurse Education Today,33(), pp.1605-1611. Australia |
| --- | --- | --- | --- | --- |
| S1. Are there clear research questions? | 2 | 2 | 2 | 2 |
| S2. Do the collected data allow to address the research questions? | 2 | 2 | 2 | 2 |
| **1. Qualitative** |  |  |  |  |
| 1.1. Is the qualitative approach appropriate to answer the research question? | 2 | 2 | 2 | 2 |
| 1.2. Are the qualitative data collection methods adequate to address the research question? | 2 | 2 | 1 | 2 |
| 1.3. Are the findings adequately derived from the data? | 2 | 2 | 2 | 2 |
| 1.4. Is the interpretation of results sufficiently substantiated by data? | 1 | 2 | 2 | 2 |
| 1.5. Is there coherence between qualitative data sources, collection, analysis and interpretation? | 2 | 1 | 1 | 1 |
| 4. **Quantitative descriptive** |  |  |  |  |
| 4.1. Is the sampling strategy relevant to address the research question? | 1 | 1 | 2 | 1 |
| 4.2. Is the sample representative of the target population? | 2 | 2 | 2 | 1 |
| 4.3. Are the measurements appropriate? | 2 | 2 | 2 | 2 |
| 4.4. Is the risk of nonresponse bias low? | 1 | 1 | 1 | 1 |
| 4.5. Is the statistical analysis appropriate to answer the research question? | 2 | 2 | 2 | 2 |
| **5. Mixed methods** |  |  |  |  |
| 5.1. Is there an adequate rationale for using a mixed methods design to address the research question? | 2 | 1 | 2 | 2 |
| 5.2. Are the different components of the study effectively integrated to answer the research question? | 2 | 2 | 2 | 2 |
| 5.3. Are the outputs of the integration of qualitative and quantitative components adequately interpreted? | 2 | 2 | 2 | 2 |
| 5.4. Are divergences and inconsistencies between quantitative and qualitative results adequately addressed? | 2 | 1 | 2 | 2 |
| 5.5. Do the different components of the study adhere to the quality criteria of each tradition of the methods involved? | 2 | 2 | 2 | 2 |
| **Judgement (low/medium/high quality)** | **31/34** | **29/34** | **31/34** | **30/34** |

###### G. JBI appraisal criteria (An observational study).

| **Study Details**  Amandu, G., Muliira, J. and Fronda, D., (2013). Using Moodle E-learning Platform to Foster Student Self-directed Learning: Experiences with Utilization of the Software in Undergraduate Nursing Courses in a Middle Eastern University. Procedia - Social and Behavioural Sciences, 93, pp.677-683. Oman | | | | |  |
| --- | --- | --- | --- | --- | --- |
| **Critical appraisal of JBI** | **Yes(1)** | **No(0)** | **Not clear(0)** | **Not applicable**  **(0)** |  |
|  |  |  |  |  |  |
| 1. Is the source of the opinion clearly identified? |  |  | 0 |  |  |
| 1. Does the source of opinion have standing in the field of expertise? | 1 |  |  |  |  |
| 3.Are the interests of the relevant population the  central focus of the opinion? | 1 |  |  |  |  |
| 1. Is the stated position the result of an analytical process, and is there logic in the opinion expressed? | 1 |  |  |  |  |
| 5.Is there reference to the extant literature? | 1 |  |  |  |  |
| 6. Is any incongruence with the literature/sources  logically defended? | 1 |  |  |  |  |
| **Total Score** | **5/6** | | | |  |

######

###### H. 1.JBI appraisal criteria (Quasi-experimental post treatment).

| **Study Details**  Sáiz-Manzanares, M., Escolar-Llamazares, M. and Arnaiz González, Á., (2020). Effectiveness of Blended Learning in Nursing Education. International Journal of Environmental Research and Public Health, 17(5), p.1589. Spain | | | | |
| --- | --- | --- | --- | --- |
| **Critical appraisal of JBI** | **Yes**  **(1)** | **No**  **(0)** | **Not clear (0)** | **No applicable (0)** |
| 1. Is it clear in the study what is the cause’ and what is the ‘effect’ (i.e. there is no confusion about which variable comes first)? |  |  | 0 |  |
| 2.Were the participants included in any comparisons similar? | 1 |  |  |  |
| 3.Were the participants included in any comparisons receiving similar treatment/care, other than the exposure or intervention of interest? | 1 |  |  |  |
| 4. Was there a control group? | 1 |  |  |  |
| 5.. Were there multiple measurements of the outcome both pre and post the intervention/exposure? | 1 |  |  |  |
| 6. Was follow up complete and if not, were differences between groups in terms of their follow up adequately described and analyzed? | 1 |  |  |  |
| 7. Were the outcomes of participants included in any comparisons measured in the same way? | 1 |  |  |  |
| 8. Were outcomes measured in a reliable way? | 1 |  |  |  |
| 9. Was appropriate statistical analysis used? | 1 |  |  |  |
| **Total Score** | **8/9** | | | |

###### H. 2.JBI appraisal criteria (Quasi-experimental post treatment).

| **Study Details**  Hemmati Malsakpak M, Pourteimour S. Comparison of the Effects of E-learning Blended with Collaborative Learning and Lecture-Based Teaching Approaches on Academic Self-Efficacy among Undergraduate Nursing Students: A Quasi-Experimental Study. J Adv Med Educ Prof. 2024;12(2):102-110. DOI: 10.30476/JAMP.2024.99100.1828..Iran. | | | | |
| --- | --- | --- | --- | --- |
| **Critical appraisal of JBI** | **Yes**  **(1)** | **No**  **(0)** | **Not clear (0)** | **No applicable (0)** |
| 1. Is it clear in the study what is the cause’ and what is the ‘effect’ (i.e. there is no confusion about which variable comes first)? | 1 |  |  |  |
| 2.Were the participants included in any comparisons similar? | 1 |  |  |  |
| 3.Were the participants included in any comparisons receiving similar treatment/care, other than the exposure or intervention of interest? | 1 |  |  |  |
| 4. Was there a control group? | 0 |  |  |  |
| 5.. Were there multiple measurements of the outcome both pre and post the intervention/exposure? | 1 |  |  |  |
| 6. Was follow up complete and if not, were differences between groups in terms of their follow up adequately described and analyzed? | 1 |  |  |  |
| 7. Were the outcomes of participants included in any comparisons measured in the same way? | 1 |  |  |  |
| 8. Were outcomes measured in a reliable way? | 1 |  |  |  |
| 9. Was appropriate statistical analysis used? | 1 |  |  |  |
| **Total Score** | **8/9** | | | |
